# Circulating Biomarkers for Prediction of Immunotherapy Response in NSCLC

**DOI:** 10.3390/biomedicines11020508

**Published:** 2023-02-09

**Authors:** Kah Yee Goh, Terence You De Cheng, Su Chin Tham, Darren Wan-Teck Lim

**Affiliations:** 1 Division of Medical Oncology, National Cancer Centre Singapore, 11 Hospital Crescent, Singapore 169610, Singapore; 2 Institute of Molecular and Cell Biology, Agency for Science, Technology and Research, Proteos, Singapore 138673, Singapore; 3 Office of Academic and Clinical Development, Duke-NUS Medical School, Singapore 169857, Singapore

**Keywords:** liquid biopsy, biomarkers, immunotherapy, checkpoint inhibition, lung cancer

## Abstract

Non-small cell lung cancer (NSCLC) constitutes the majority of the lung cancer population and the prognosis is poor. In recent years, immunotherapy has become the standard of care for advanced NSCLC patients as numerous trials demonstrated that immune checkpoint inhibitors (ICI) are more efficacious than conventional chemotherapy. However, only a minority of NSCLC patients benefit from this treatment. Therefore, there is an unmet need for biomarkers that could accurately predict response to immunotherapy. Liquid biopsy allows repeated sampling of blood-based biomarkers in a non-invasive manner for the dynamic monitoring of treatment response. In this review, we summarize the efforts and progress made in the identification of circulating biomarkers that predict immunotherapy benefit for NSCLC patients. We also discuss the challenges with future implementation of circulating biomarkers into clinical practice.

## 1. Introduction

Lung cancer is the leading cause of cancer-related mortalities, with approximately 1.7 million deaths per year globally [1,2]. In the United States, estimated deaths from lung cancer top the chart, making up 21% of all cancer-related deaths in both sexes [3]. There are two main types of lung cancer, non-small cell lung cancer (NSCLC) and small cell lung cancer (SCLC). NSCLC constitutes the majority (about 85%) of the lung cancer population [1] and is further classified into adenocarcinoma, squamous cell carcinoma and large cell carcinoma [4,5]. The prognosis for NSCLC is extremely poor due to late-stage diagnosis and resistance to therapeutic treatments [6,7,8]. The five year survival rate for NSCLC is around 15–18% [9,10,11].

Conventional treatment modalities for NSCLC include surgery, chemotherapy and radiation therapy. In the past decade, the introduction of targeted therapy and immunotherapy has tremendously advanced the treatment landscape of NSCLC [12,13]. The choice of treatment largely depends on the stage of disease and molecular characteristics of the tumor. For early stage NSCLC patients (stage I–II), surgery and/or radiation therapy are the primary approaches [14]. For locally advanced NSCLC patients (stage III) with resectable tumors, surgery and chemoradiotherapy are recommended, while for the unresectable cases, chemoradiotherapy and immunotherapy are recommended [14]. For metastatic NSCLC patients (stage IV), targeted therapy is typically used for patients whose tumors carry driver gene mutations (e.g., EGFR, ALK, MET, ROS1, KRAS) but they are only present in a small proportion of NSCLC patients (around 25%) [10,14,15]. The vast majority of advanced NSCLC patients lack actionable driver gene mutations and thus for this group of patients, they are often treated with a combination of chemoradiotherapy and immunotherapy [14].

Immunotherapy leverages on the patient’s immune system to recognize and kill tumor cells. In the context of NSCLC, immune checkpoint inhibitors (ICI) were first approved by the FDA in 2015 [16]. In the last several years, numerous trials conducted on advanced NSCLC patients demonstrated that the use of ICI alone or in combination with chemotherapy resulted in more durable responses, longer survival and better overall response rates compared to the use of chemotherapy agents alone [17,18,19,20,21,22,23]. Specifically, the five year survival for advanced NSCLC patients treated with ICI had at least a two-fold increase in survival rate compared to the chemotherapy group [24,25,26]. Therefore, ICI has become a standard of care for advanced NSCLC patients, either as monotherapy or in combination with chemotherapy [27,28].

However, ICI treatments are costly (up to USD 12,000 per month) [29], thus not all patients can afford the hefty treatment. Additionally, ICI does not benefit all advanced NSCLC patients [28]; only a minority of NSCLC patients (<20%) respond to ICI treatment [30,31,32,33,34,35]. Immune-related adverse effects such as inflammatory arthritis, colitis, pneumonitis, thyroiditis and nephritis were also reported in patients treated with ICI [36,37]. The overall responses are highly variable and influenced by individual tumor characteristics such as tumor PD-L1 expression (also known as Tumor Proportion Score; TPS), tumor mutation burden (TMB), tumor infiltrating lymphocytes (TILs), host immune system and molecular signatures [21,38,39,40]. Therefore, prediction of immunotherapy response is crucial to identify patients who will most likely benefit from the expensive therapeutic and avoid subjecting patients who do not benefit from ICI to the unnecessary adverse effects. In this review, we summarize biomarkers that can predict responses to ICI in NSCLC patients, with a particular focus on circulating biomarkers present in the peripheral blood. We also outline the challenges ahead with implementation of these circulating biomarkers into clinical practice.

## 2. Mechanism of Immune Checkpoint Inhibition

To understand how the immune checkpoint functions, we first describe how normal T lymphocytes are activated. The activation of naïve T cells requires at least two signals: (i) antigen recognition signal, where T cell receptors (TCR) expressed on the T cell surface must recognize and bind to the pathogenic antigen presented by the Major Histocompatibility Complex (MHC) on the surface of antigen presenting cells (APCs), (ii) co-stimulatory signal, where CD28 expressed on the T cell surface must interact with the B7 (CD80 and CD86) molecule expressed on the surface of APCs [41]. If the antigen recognition signal is weak due to low levels of antigens, an additional third signal is required for complete T cell activation [42]. The third signal is the cytokine polarizing signal, where APCs release inflammatory cytokines (e.g., IL-12 or type I IFN) that bind to respective cytokine receptors on T cells to stimulate T cell expansion and differentiation [43].

Immune checkpoints are self-tolerance mechanisms that prevent T lymphocytes from destroying one’s own cells during an inflammatory response, protecting oneself from auto-immune attacks. The immune checkpoints are governed by cell surface receptors present on T lymphocytes including PD1, CTLA4, TIM3, LAG3, TIGIT and VISTA [44]. The binding of respective ligands to these checkpoint receptors mediate immune suppression. To date, PD1 and CTLA4 checkpoint receptors are most extensively studied in the context of immunotherapy while other checkpoint receptors are still undergoing clinical investigations [44].

PD1 and CTLA4 suppress T cell activation by distinct mechanisms [45,46]. In the PD1 pathway, binding of the cognate ligands, PD-L1 or PD-L2, to PD1 receptor on antigen-stimulated T cells inhibits T cell proliferation and activation [47]. In cancer patients, tumor cells exploit this immune checkpoint by upregulating PD-L1 expression to enable interaction with PD1 on T cells, leading to immune suppression [48,49,50]. This allows tumor cells to escape immunosurveillance and promote their survival. On the other hand, the CTLA4 receptor is a CD28 homolog, which has a higher binding affinity to B7 molecule than CD28 [51,52,53]. Therefore, CTLA4 blocks the interaction of CD28 and B7, directly interfering with the costimulatory signal required for T cell activation. In cancer patients, tumor cells exploit this immune checkpoint by the constitutive expression of CTLA4, which binds to the B7 molecule and blocks the costimulatory signal, thereby inhibiting T cell activation [54].

The ICI drugs are designed to block PD1/PD-L1 or CTLA4/B7 pathway interaction, relieving the inhibitory effects on T cells, allowing re-activation, proliferation and differentiation of T cells, enhancing the immune function and anti-tumor response. Since 2015, multiple ICI drugs have been approved by the FDA for clinical use in NSCLC patients, either as the first- or second-line of treatment (Table 1). The FDA-approved ICI drugs applied to NSCLC patients include nivolumab, pembrolizumab, atezolizumab, durvalumab, cemiplimab, ipilimumab and tremelimumab. Of these, only ipilimumab and tremelimumab are targeting the CTLA4 pathway; the rest are targeting the PD1/PD-L1 signaling axis.

## 3. Tissue Biopsy Versus Liquid Biopsy

Tissue biopsy has always been the gold standard for clinical diagnosis and evaluation. Since immunotherapy only benefits a minority of advanced NSCLC patients, predictive biomarkers are actively developed to forecast the benefit of ICI. The prominent tumor-related biomarkers include tumor PD-L1 (TPS), TMB, TILs, microsatellite instability (MSI), tumor mismatch repair deficiency (dMMR) and tumor transcriptomic signatures. Of these, tumor PD-L1, TMB and MSI were successfully developed into FDA-approved companion diagnostic assays (PD-L1 IHC that measures TPS and Foundation One CDx panel that evaluates TMB and MSI). They are recommended for patients who intend to receive immunotherapy as a means to predict the ICI benefit and help clinicians in deciding the optimal treatment strategy. NSCLC patients with high tumor PD-L1 expression (TPS ≥ 50%) or high TMB (≥10 mutations per Mb) would typically be recommended for ICI monotherapy.

The existing companion diagnostic assays require testing on tissue biopsy. The availability and accessibility to tissue biopsies limit the use of such assays. Patients who lack tissue biopsy may not stand a chance to receive immunotherapy treatment. Furthermore, tumor biopsies are known to be heterogeneous, thus the evaluation of PD-L1 expression, TMB or MSI can be variable depending on the section obtained [71,72]. More importantly, the performance of tumor biomarkers in predicting ICI response is not robust enough because not all NSCLC patients with high PD-L1 expression or high TMB are responders and there are some patients with low PD-L1 or low TMB who benefitted from anti-PD1/PD-L1 therapy [62,73,74].

Given the limitations of tumor biomarkers in predicting ICI response, numerous studies are looking for more effective biomarkers. The circulating biomarkers present in peripheral blood are currently under intensive research. Liquid biopsy (through blood sampling) offers several advantages over tissue biopsy. First, blood collection is non-invasive, less expensive and more readily accessible than a tissue biopsy. Second, blood collection allows repeated sampling, which enables the dynamic and real-time monitoring of ICI response and resistance throughout the course of treatment whereas tissue biopsy is usually performed one time at pre-treatment. Third, liquid biopsy can overcome the spatial and temporal heterogeneity that is associated with tissue biopsy.

## 4. Circulating Biomarkers

**Figure 1:**
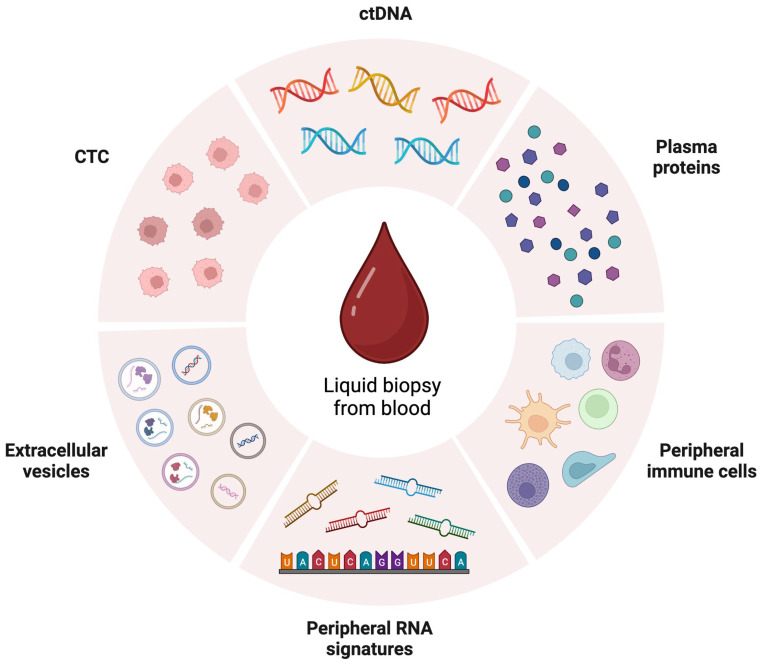
Circulating analytes present in the peripheral blood that are used for liquid biopsy. The image was created with BioRender.com. Abbreviations: CTC, circulating tumor cells; ctDNA, circulating tumor DNA.

The peripheral blood carries multiple components including white blood cells, platelets, plasma proteins and vesicles that may play a role in monitoring treatment response or resistance (Figure 1). To date, the FDA has approved two plasma-based NGS assays (Guardant360 and Foundation One Liquid CDx) that detect the presence of actionable driver gene mutations in the circulating tumor DNA of NSCLC patients. However, these two assays are only approved as companion diagnostics specifically to guide the selection of targeted therapy (e.g., EGFR, ALK, MET inhibitors). The FDA has not approved any circulating biomarkers for the prediction of immunotherapy response. We examined the potential of how each circulating analyte and biomarker associate with ICI response and summarize their respective advantages and limitations (Table 2).

### 4.1. Circulating Tumor DNA (ctDNA)

Circulating tumor DNA (ctDNA) are DNA fragments shed by tumor cells and released into the peripheral blood, usually present in the plasma or serum. ctDNA is a small subset of cell-free DNA (cfDNA), comprising about 5–10% of the total pool of cfDNA in late-stage cancer patients [75,76]. ctDNA is primarily distinguished from non-malignant cfDNA by the shorter fragment size (around 50–150 bp) and presence of characteristic genetic mutations associated with the cancer [76]. ctDNA detection and analysis are performed by several methods including next-generation sequencing (NGS), real time quantitative PCR (qPCR) and droplet digital PCR [77]. Of all the circulating biomarkers that predict ICI response, ctDNA is most extensively studied in terms of ctDNA level, tumor genetic alterations and blood TMB.

Several studies found that the level of ctDNA correlates with ICI treatment response and resistance in NSCLC patients. A prospective pilot study in NSCLC, melanoma and colorectal cancer patients treated with nivolumab or pembrolizumab monotherapy showed that patients with undetectable ctDNA at 8 weeks post-treatment presented a marked response to anti-PD1 blockade, with longer progression-free survival (PFS) and overall survival (OS) [78]. Longitudinal analysis of ctDNA level in 28 metastatic NSCLC patients treated with ICI demonstrated that >50% reduction of ctDNA level from baseline was associated with superior OS (HR = 0.17, *p* = 0.007). This suggests that a decrease in ctDNA level is an early marker for therapeutic efficacy in ICI-treated NSCLC patients [79]. The association of ctDNA level to anti-PD1/PD-L1 response was further supported by other studies that examined baseline and post-treatment changes in ctDNA level of NSCLC patients [80,81,82,83,84,85].

ctDNA is used to detect tumor-specific genetic alterations including somatic mutations and DNA methylation patterns, especially in patients with insufficient tissue biopsy. In 86 ICI-treated NSCLC patients with evaluable baseline ctDNA, the presence of PTEN or STK11 mutation was correlated with early disease progression (HR = 8.9, *p* = 0.09 for PTEN; HR = 4.7, *p* = 0.003 for STK11) while transversion mutations (conversion between purine and pyrimidine) in KRAS and TP53 predicted better clinical outcomes (HR = 0.36, *p* = 0.011 for TP53; HR = 0.46, *p* = 0.11 for KRAS) [86]. Co-mutations in KRAS/STK11 and KRAS/STK11/TP53 negatively impact the OS and PFS of ICI-treated NSCLC patients [87]. In another study, atezolizumab-treated NSCLC patients with KEAP1/NFE2L2 mutations had significantly poorer OS compared to patients with wild type KEAP1/NFE2L2 (HR = 1.97, 95% CI = 1.48–2.63, *p* < 0.001) [88]. Therefore, the presence of STK11 and KEAP1/NFE2L2 mutations correlated with a lack of clinical benefit in ICI-treated NSCLC patients [27,86,87,88].

Aberrant DNA methylation patterns are also commonly found in NSCLC tumors and can be detected from ctDNA. DNA methylation are typically found at CpG islands at the promoter region or within the first exon of mammalian genes [89]. Hypermethylation usually occurs at tumor suppressor genes and hypomethylation occurs at oncogenes, resulting in transcriptional repression and activation respectively [90,91]. Notably, methylation patterns are stable and specific to cell types, allowing identification of the tissue origin for ctDNA [89], which is especially useful for characterization in metastatic cancer patients. In several studies of NSCLC patients treated with anti-PD1 inhibitor, responders exhibited methylation patterns on cis-regulatory elements (promoters and enhancers) of specific genes that were distinct from non-responders [34,92,93]. The unmethylated status of FOXP1 and hypomethylation status of CYTIP and TNFSF8 were associated with improved anti-PD1 response, PFS and OS [92,93]. Of note, these DNA methylation patterns were observed in tumor specimens and not plasma-derived ctDNA. An observational study on plasma-derived ctDNA from NSCLC patients receiving immunotherapy or chemoradiotherapy showed that a combined decrease in tumor mutations and methylated DNA associated with better OS [94]. Therefore, consistent with the aforementioned DNA methylation studies on tumor tissues, hypomethylation associates with better response to ICI treatment in NSCLC patients.

ctDNA also represents an alternative measure for TMB. As opposed to tissue-derived TMB (tTMB), ctDNA-derived TMB is termed as blood TMB (bTMB), which is typically measured by NGS analysis on a targeted gene panel. Apart from the Foundation One CDx panel used on tumor tissues to measure tTMB, the FDA also approved a Foundation One Liquid CDx panel, covering 394 genes that can be applied to blood plasma to detect mutations, bTMB and MSI. However, unlike the tumor Foundation One CDx panel, the Foundation One Liquid CDx panel is indicated as a companion diagnostic specifically for targeted therapies in NSCLC (tyrosine kinase inhibitors against EGFR, ALK, MET) and not yet applicable to immunotherapy.

Gandara et al. used the Foundation One Liquid CDx panel to measure bTMB in a retrospective analysis of two large clinical trials (POPLAR and OAK). They found that bTMB is a predictor of clinical benefit in NSCLC patients treated with atezolizumab [95]. NSCLC patients with high bTMB (≥16 mutations/Mb) were associated with improved response rate and PFS from atezolizumab given as a second line or higher setting [95]. An analysis of 48 advanced NSCLC patients with matched blood and tumor tissues revealed that bTMB (obtained from a sequencing panel of 150 cancer-related genes) correlated well with tTMB (obtained from whole exome sequencing) (spearman correlation = 0.62; median r2 = 0.91; interquartile range, 0.89–0.92) [96]. In the anti-PD1/PD-L1 treatment group, NSCLC patients with bTMB ≥ 6 mutations/Mb were associated with better PFS and objective response rates (ORR) [96]. In another similar study, Chen et al. evaluated the role of bTMB and tTMB in 56 advanced NSCLC patients receiving ICI using a larger sequencing panel of 520 cancer-related genes. They also found a positive correlation between bTMB and tTMB. NSCLC patients with high bTMB (≥11 mutations/Mb) had significantly longer PFS than patients with low bTMB (<11 mutations/Mb) [97].

In the MYSTIC phase 3 trial, among 809 metastatic NSCLC patients with evaluable bTMB based on a panel of 500 genes, only those with bTMB ≥ 20 mutations/Mb showed improved OS for the dual checkpoint blockade of durvalumab plus tremelimumab (median OS = 21.9 months; 95% CI = 11.4–32.8) versus chemotherapy (median OS = 10.0 months; 95% CI = 8.1–11.7) [98]. In the B-F1RST phase 2 trial with 152 advanced NSCLC patients receiving first-line atezolizumab monotherapy, patients with bTMB ≥ 16 (defined as 14.5 mutations/Mb) had higher ORR and the ORR improved further with higher bTMB cutoffs [99]. Although multiple studies highlighted the promising role of bTMB as a predictive biomarker for ICI response, not all studies support this. For example, Chae et al. showed that among 20 NSCLC patients treated with ICI, higher bTMB was significantly correlated with shorter OS and PFS [100]. The bTMB cutoff and gene panel used were not standardized across studies, leading to different interpretations of results.

Since ctDNAs possess several features (ctDNA level, bTMB, mutations) that could each predict response to ICI treatment, some studies explored how the combination of ctDNA features may correlate to ICI response. Aggarwal et al. found that metastatic NSCLC patients with baseline bTMB ≥ 16 mutations/Mb and an absence of negative predictor mutations (STK11/KEAP1/PTEN and HER2 exon 20) were associated with improved PFS and durable clinical benefit in response to first line pembrolizumab treatment [101]. Nabet et al. examined a ctDNA-normalized bTMB score (based on the ratio of ctDNA level and bTMB) in relation to ICI response. They found that ICI-treated patients with higher normalized bTMB scores had significantly better clinical outcomes and more durable responses (PFS HR = 1.46, *p* = 0.001) [102].

### 4.2. Circulating Tumor Cells (CTCs)

CTCs are cells disseminated from the primary or metastatic tumor into the peripheral blood. They are approximately 12–25 μm [103] and present in extremely low numbers (typically 1–10 CTCs per 10 mL of blood or ~1–100 CTCs per 10^9^ blood cells) [104,105,106]. CTCs are present as single cells or in clusters in the peripheral blood of NSCLC patients [107,108]. They carry valuable genetic, transcriptomic and proteomic information that may aid in diagnosis, treatment and prognosis.

The relationship between CTC number and response to ICI therapy has been explored. A high number of CTCs present at baseline prior to ICI initiation was associated with poorer prognosis and a higher risk of disease progression in advanced NSCLC patients [109,110,111]. Apart from CTC numbers, several groups also analyzed the correlation between PD-L1 expression on CTCs and ICI response. In 24 nivolumab-treated NSCLC patients, Nicolazzo et al. found that the presence of PD-L1+ CTCs at baseline, 3 months and 6 months post-treatment correlated with poor outcomes and disease progression [110]. This observation was similarly reported in other independent studies [109,112,113]. An analysis of 96 NSCLC patients receiving second-line nivolumab revealed that higher baseline PD-L1+ CTCs was observed in non-responders who progressed within 6 months of treatment [109]. An increase in PD-L1+ CTCs through the course of treatment was associated with resistance to PD1/PD-L1 inhibitors in NSCLC patients [113]. Besides PD-L1, other immune biomarkers expressed by CTCs are also under investigation. For instance, Papadaki et al. showed that the presence of indoleamine-2,3-dioxygenase IDO+ CTCs was associated with shorter PFS and OS in NSCLC patients treated with PD1 inhibitors [111].

Of note, the correlation between expression of PD-L1 on CTC and matched tumor biopsy remains controversial. Ilie et al. reported a 93% concordance between CTC PD-L1 expression and matched tumor PD-L1 among 106 NSCLC patients [114]. In contrast, other groups reported no significant correlation between CTC PD-L1 expression and tumor PD-L1 expression in NSCLC patients [109,113,115]. The discrepancy is likely due to differences in CTC isolation, enrichment, analysis and patient cohorts.

### 4.3. Peripheral Immune Cells

The success of immunotherapy is highly dependent on the host’s ability to mount an anti-tumor immune response. Therefore, the host’s immune status greatly influences the outcome of immunotherapy that is administered systemically. An increasing number of studies focused on how peripheral immune cell populations affect an individual’s response to ICI treatment. Here, we describe how CD8 T cells, CD4 T cells, regulatory T cells, TCR repertoire and the neutrophils-to-lymphocytes ratio (NLR) affect ICI response in NSCLC patients.

Based on 74 advanced NSCLC patients treated with nivolumab, a higher density of CD4+ and CD8+ T cells was observed in responders at baseline [116], suggesting that a higher circulating T lymphocyte level may predict response to ICI. Moreover, responders had a significantly lower baseline expression of PD1 on CD8+ T cells compared to non-responders [116]. In another study of 29 advanced NSCLC patients treated with anti-PD1 inhibitors, an increase in the proliferation of PD1+ CD8+ T cells levels was observed in responders within the first two cycles of immunotherapy [117]. The proliferating PD1+ CD8+ T cells exhibited an effector-like phenotype (HLA-DR+, CD38+, Bcl-2low) and expressed costimulatory molecules (CD28, CD27, ICOS) [117]. The proliferative PD1+ CD8+ T cells phenotype was also observed by Kim et al., as early as one week after PD1-targeted therapy initiation in epithelial thymic carcinoma and NSCLC patients [118].

Besides the proliferative PD1+ CD8+ T cells phenotype, responders also had a significantly lower baseline frequency of exhausted T cells (characterized by CD8+ PD1+ Eomes+) compared to non-responders [116]. Longitudinal analysis of blood samples revealed the frequency of exhausted T cells continued to decrease during the course of treatment [116]. Additionally, Ferrara et al. showed that the presence of baseline senescent CD8+ T cell population (characterized by low proliferation, CD28-, CD57+, KLRG+) was associated with shorter PFS, shorter OS and lower objective responses in NSCLC patients treated with ICI [119].

Regulatory T cells (Tregs) are a subset of CD4+ T cells that maintain the homeostasis of immune system and prevent autoimmune attacks by having immune-suppressive functions. A study of 28 NSCLC patients treated with ICI combination therapy found that patients with lower level of baseline Tregs experienced a better response. A higher baseline ratio of CD4+/CD8+ T cell predicted longer PFS to ICI treatment [120]. Using a discovery cohort of 83 NSCLC patients and a validation cohort of 49 NSCLC patients treated with anti-PD1 inhibitors, Koh et al. found that responders had a higher frequency of circulating Tregs (characterized by CD25+ FOXP3+ CD4+ T cells) than non-responders one week after treatment initiation. The high level of circulating Tregs at post-treatment was correlated with longer PFS and OS [121].

The NLR refers to the absolute neutrophil count divided by the absolute lymphocyte count in peripheral blood. It is a marker for general immune response and tumor-related inflammation. The NLR has been widely explored for correlation to ICI response, as the information is readily available from a routine blood test. Several pooled meta-analyses demonstrated that high baseline NLR correlated with shorter PFS and OS in NSCLC patients treated with PD1/PD-L1 inhibitors [122,123,124]. Consistent with meta-analyses, a multicentre retrospective analysis of 119 NSCLC patients treated with second-line pembrolizumab found that patients with baseline NLR > 5 associated significantly with shorter PFS of 6.86 months (95% CI = 5.81–7.90) compared to those with NLR ≤ 5 of 18.82 months (95% CI = 15.87–21.78) (log rank *p* < 0.001) [125]. Multivariate analysis further revealed that baseline NLR > 5 was an independent predictive factor for shorter PFS (HR = 4.47, 95% CI = 2.20–9.07, *p* < 0.001), suggesting that NLR ≤ 5 is a potential predictive marker for ICI response [125]. Another retrospective study of 187 advanced NSCLC patients treated with nivolumab (second line or later) reported the same trend, in which baseline NLR ≤ 5 was associated with improved PFS (*p* = 0.028) and OS (*p* = 0.001) [126]. Ayers et al. further confirmed that high NLR (>5) was associated with poorer OS (HR = 1.66; *p* = 0.019) in metastatic NSCLC patients treated with nivolumab, atezolizumab or pembrolizumab at any line of therapy [127]. A sustained high NLR at 2–8 weeks after ICI initiation had a greater impact on survival outcomes than baseline NLR (HR = 3.43; *p* = 4.23 × 10^−8^), regardless of PD-L1 TPS status [127]. In another cohort of 221 metastatic NSCLC patients receiving first-line pembrolizumab, Alessi et al. defined the optimal baseline NLR cutoff to distinguish responders from non-responders. Patients with baseline NLR < 2.6 had a significantly higher ORR (52.4% vs. 24.7%, *p* < 0.001), longer median PFS (10.4 vs. 3.4 months, HR = 0.48, 95% CI = 0.35–0.66, *p* < 0.001) and longer median OS (36.6 vs. 9.8 months, HR = 0.34, 95% CI = 0.23–0.49, *p* < 0.001) compared to patients with NLR ≥ 2.6 [128].

In the majority of T cells, TCR consists of one alpha and one beta chain [129]. Each chain has three hyper-variable complementary-determining regions (CDRs). The variability in CDR3 is generated by V(D)J recombination and confers a unique sequence to each TCR [130]. Thus, sequencing analysis of this specific region reveals the TCR repertoire, which represents the total number of TCRs expressed on T cells of an individual, reflecting the diversity and clonality of TCR. In recent years, the peripheral TCR repertoire has been shown to associate with ICI response. Han et al. collected pre- and post-ICI blood samples from 40 NSCLC patients treated with anti-PD1/PD-L1 therapy. Patients with high pre-treatment PD1+ CD8+ TCR diversity had better response and longer PFS than patients with low diversity (6.4 months vs. 2.5 months, HR = 0.39; 95% CI = 0.17–0.94; *p* = 0.021) [131]. Patients with an increased PD1+ CD8+ TCR clonality after ICI treatment had longer PFS than those with reduced clonality (7.3 months vs. 2.6 months, HR = 0.26; 95% CI = 0.08–0.86; *p* = 0.002) [131]. In line with this study, a prospective analysis of 12 advanced NSCLC patients treated with second-line atezolizumab found that responders had an increase in CD8+ TCR diversity during the course of treatment [132]. Contrary to these studies, Nakahara et al. showed that among 20 EGFR/ALK-wild type NSCLC patients treated with anti-PD1 therapy, responders had a significant reduction in TCR diversity 6 weeks after treatment initiation compared to non-responders [133]. Therefore, the role of TCR repertoire in predicting ICI response remains debatable. The differences are likely due to a lack of standardization in TCR sequencing and bioinformatics analysis pipelines.

### 4.4. Extracellular Vesicles

Extracellular vesicles (EVs) are small membrane-bound vesicles secreted by cells into the extracellular space and peripheral blood. They carry complex cargos such as proteins, nuclei acids and lipids that are involved in cell–cell communication. There are three types of EVs, exosomes, microvesicles and apoptotic bodies. Exosomes are the most well characterized and constitute the largest group of EVs. In the context of immunotherapy, we focused on how the cargo contents of exosomes relate to ICI response, specifically exosomal PD-L1 and miRNAs.

Within a cohort of 30 advanced EGFR/ALK-wild type NSCLC patients treated with anti-PD1/PD-L1, non-responders had a significantly higher expression of exosomal miR-320b, -320c and -320d than responders at baseline [134]. The down-regulation of exosomal miR-125b-5p during treatment was associated with better response to ICI [134]. In another cohort of 29 advanced NSCLC patients treated with anti-PD1/PD-L1 monotherapy, Shukuya et al. identified seven EV-associated miRNAs that had significant concentration differences between responders and non-responders at baseline. miR-1246-5p and miR-378c-5p were more highly expressed in responders, while miR-1296-5p, miR-4707-3p, miR-1229-3p, miR-874-3p and miR-1468-5p were more highly expressed in non-responders [135]. More recently, an analysis of pre-treatment plasma EV-associated miRNAs in 88 advanced NSCLC patients receiving anti-PD1 monotherapy revealed that high pre-treatment expression of EV-miR-625-5p was significantly associated with better objective response and longer OS [136].

Some groups have investigated the relationship between exosomal PD-L1 expression and immunotherapy efficacy. Exosomal PD-L1 protein expression was quantitated at the surface of exosomes using ELISA, immunoblot or flow cytometry. Analysis of blood samples from 149 ICI-treated NSCLC patients demonstrated that responders had significantly lower expression of exosomal PD-L1 protein than non-responders at pre-treatment [137]. An increase in exosomal PD-L1 protein expression 3–6 weeks after ICI treatment was associated with better disease control rate (DCR) and longer PFS [137]. Similar results were reported by another study, in which increased expression of exosomal PD-L1 protein after two months of ICI treatment predicted better PFS, OS and ORR in metastatic NSCLC patients [138]. Miguel-Perez et al., however, reported the opposite trend. From a retrospective cohort of 33 NSCLC patients and prospective cohort of 39 NSCLC patients, they found that non-responders had an increase in EV PD-L1 protein expression at 9 weeks post-treatment compared to baseline [139]. The discrepancy is likely due to differences in the exosome isolation method, PD-L1 protein quantification method and patient cohorts.

The correlation between circulating exosomal PD-L1 and tumor PD-L1 remains controversial. Kim et al. found a correlation between the number of plasma-derived PD-L1+ exosomes and tumor PD-L1 expression in a small study of 24 NSCLC patients [140]. Shimada et al. also reported a significant correlation between serum-derived exosomal PD-L1 level and tumor PD-L1 level in a larger study of 120 NSCLC patients [141]. However, Li et al. did not observe any correlation between serum-derived exosomal PD-L1 and tumor PD-L1 expression in a cohort of 85 NSCLC patients [142]. It is important to note that exosomes can be secreted by any cell type including tumor cells, immune cells and stromal cells. Therefore, it is difficult to ascertain the cell type origin of exosomes, and this may partially explain why exosomal PD-L1 expression does not correlate well with tumor PD-L1 expression.

### 4.5. Plasma Cytokines and Chemokines

Cytokines and chemokines are soluble proteins present in the peripheral blood, secreted by immune cells (e.g., monocytes, macrophages, neutrophils, lymphocytes) and non-immune cells (e.g., endothelial cells, epidermal cells and fibroblasts). They bind to their cognate receptors on the surface membrane of target cells, trigger intracellular signaling and modulate growth and activity of these cells [143,144].

Multiple studies explored the relationship between plasma cytokines and ICI response, particularly on the proinflammatory cytokines. An analysis of 47 NSCLC patients showed that patients with a decreasing level of IL-6 after ICI treatment experienced better PFS than those with stable or increasing level of IL-6 (11 vs. 4 months, HR = 0.45; 95% CI = 0.23–0.89, *p* = 0.04) [145]. In addition, Kang et al. examined the baseline serum level of IL-6 in 125 advanced NSCLC patients treated with PD1/PD-L1 inhibitors. They found that patients with low IL-6 (<13.1 pg/mL) had a higher ORR and DCR than those with high IL-6 (ORR 33.9% vs. 11.1%, *p* = 0.003; DCR 80.6% vs. 34.9%, *p* < 0.001). The low IL-6 group had significantly longer median PFS and OS than the high IL-6 group [146]. In agreement with previous studies, a prospective analysis of cytokine profile in 29 metastatic NSCLC patients receiving second line anti-PD1 monotherapy revealed that patients with higher IL-6 levels had a less durable response and significantly poorer PFS (5.14 vs. 38.57 weeks; *p* < 0.001) compared to patients with lower IL-6 levels [147].

Sanmamed et al. evaluated changes in serum IL-8 levels at baseline and post-treatment in 19 NSCLC patients treated with nivolumab or pembrolizumab. Responders had a decrease in IL-8 level while non-responders had an increase in IL-8 level, as early as 2–4 weeks after treatment initiation. An early decrease in serum IL-8 level was associated with longer OS in NSCLC patients [148]. Two other studies also reported similar findings, in which ICI-treated NSCLC patients with increased level of plasma IL-8 experienced poorer survival [147,149]. A large retrospective analysis of 1344 patients with melanoma, NSCLC or renal cell carcinoma showed that high baseline serum IL-8 (≥23 pg/mL) was associated with poorer OS and clinical outcomes in response to nivolumab and/or ipilimumab, everolimus or docetaxel treatment [150]. Therefore, lower levels of plasma IL-6 and IL-8 appear to be predictive of immunotherapy response.

Apart from interleukins, plasma IFN-gamma level was also widely studied in relationship to ICI response. Costantini et al. collected plasma from 43 NSCLC patients receiving nivolumab at baseline and the response assessment (2 months post-treatment). They found that IFN-gamma levels showed no correlation to ICI response [151]. In contrast, Hirashima et al. analyzed a group of 29 NSCLC patients receiving ICI treatment and observed that patients with low levels of IFN-gamma (<10 IU/mL) tend to experience poor response and early disease progression [152]. Consistent with these results, Kauffmann-Guerrero et al. showed that elevated level of IFN-gamma was found in NSCLC patients with a significant durable response to ICI treatment [147]. Therefore, high levels of plasma IFN-gamma appear to predict a better efficacy in ICI response.

Recent studies also interrogated the relevance of circulating chemokines to anti-PD1/PD-L1 response. A prospective study of 32 lung cancer patients treated with ICI showed that patients with high baseline plasma CXCL10 concentration experienced significantly shorter PFS than patients with low CXCL10 concentration [153]. An evaluation of the pre-treatment plasma from 43 NSCLC patients treated with ICI revealed that high plasma levels of CXCL9 and CXCL10 were significantly associated with better response and longer PFS [154]. Contrary to these findings, Harel et al. examined baseline and on-treatment plasma proteomic profiles of 143 ICI-treated NSCLC patients and found that non-responders had higher levels of plasma CXCL8 and CXCL10 at baseline and on-treatment compared to responders [155]. The apparent disparity in the association of CXCL10 with ICI response likely stems from the different methods used to quantify protein expression (flow cytometry, proximity extension assay, ELISA), different cutoffs used and patient cohorts.

### 4.6. Peripheral RNA Signatures

With continuous improvements in NGS technologies, profiling of transcriptomic landscape in the peripheral blood has become simpler and easier to accomplish. Numerous studies explored how circulating RNAs derived from plasma, serum and leukocyte lineages correlate to immunotherapy response.

Halvorsen et al. examined the pre-treatment serum miRNA profile of 51 NSCLC patients treated with nivolumab. They identified a baseline 7-miR signature (miR-93-3p, miR-215-5p, miR-411-3p, miR-493-5p, miR-494-3p, miR-495-3p, miR-548j-5p) that was significantly associated with OS > 6 months [156]. Another study demonstrated that a 24-miR signature classifier (MSC) derived from baseline plasma of 140 ICI-treated NSCLC patients could distinguish responders from non-responders. The MSC risk level was associated with ORR (*p* = 0.0009), PFS [multivariate HR = 0.31; 95% CI = 0.17–0.56; *p* = 0.0001], and OS (multivariate HR = 0.33; 95% CI = 0.18–0.59; *p* = 0.0002). In patients with responsive or stable disease, the MSC risk level decreased or remained low during treatment until disease progression [157]. An analysis of 34 NSCLC patients treated with nivolumab demonstrated that responders had increased serum expression of 10 miRNAs including miR-93, miR-138-5p, miR-200, miR-27a, miR-424, miR-34a, miR-28, miR-106b, miR-193a-3p and miR-181a from pre-treatment to post-treatment, compared to non-responders. High expression of these 10 miRNAs was significantly correlated to improvements in OS and PFS [158]. A recent study focused on the significance of immunoregulatory circulating miRNAs in predicting anti-PD1 response [159]. Using a cohort of 69 advanced NSCLC patients treated with second line nivolumab, the authors interrogated the expression level of six miRNAs involved in immune checkpoint regulation (miR-34a, miR-200b, miR-200c), T-cell activity (miR-155), function of myeloid-derived suppressive cells (miR-223) or regulatory T lymphocytes (Tregs) (miR-146a) in the pre-treatment plasma samples. The expression of four miRNAs (miR-34a, miR-146a, mir-200c, and miR-223) was associated with response to nivolumab. In the non-squamous NSCLC subgroup, high miR-200c and low miR-34a expression was significantly associated with poorer OS. In the squamous NSCLC subgroup, low miR-146a and low miR-223 expression was significantly associated with poorer DCR [159].

In addition to plasma or serum-derived miRNAs, RNA signatures from other blood components also predict response to immunotherapy. The white blood cell (WBC) RNA signatures are thought to be more representative of the host’s systemic immune status [160]. Rajakumar et al. performed whole blood miRNA profiling in 334 stage IV NSCLC patients and identified a circulating myeloid-derived miRNA signature that is predictive of OS following immunotherapy. The signature comprises five miRNAs (miR-2115-3p, miR-218-5p, miR-224-5p, miR-4676-3p, miR-6503-5p) whose expressions are used to define the miRisk score. The miRNAs expression largely originated from monocytes and neutrophils (miR-2115-3p, miR-4676-3p and miR-6503-5p), and platelets (miR-224-5p). Only miR-218-5p demonstrated heterogeneous expression in multiple cell types. Responders had a lower expression of the miRisk miRNAs, which may reflect reactivation of the PD1/PD-L1 signaling pathway in peripheral immune cells, and this reactivation status may be maintained upon migration of the peripheral immune cells into the tumor microenvironment. More importantly, the miRisk score outperformed tissue-based PD-L1 IHC in predicting response and OS to PD1 inhibitor monotherapy [160]. Rajakumar et al. further investigated the 5-miR signature in relationship to PD-L1 TPS status in a cohort of 124 stage IV NSCLC patients [161]. They prospectively collected whole blood samples from patients with PD-L1 TPS ≥ 50% before anti-PD1 therapy initiation and performed small RNA sequencing. The revised 5-miR risk score (miRisk) stratified ICI-treated patients with high PD-L1 TPS (≥50%) and identified a high-risk group with significantly shorter OS (HR = 5.24, 95% CI = 2.17–12.66, *p* < 0.001). Therefore, the miRisk score distinguishes a group of NSCLC patients within the cohort of high PD-L1 TPS, who are less likely to benefit from immunotherapy [161]. This group of patients may be given other treatment modalities to avoid the unnecessary side effects of immunotherapy.

### 4.7. Integration of Circulating Biomarkers

To further improve the prediction of ICI response, some groups looked at the integration of two or more circulating biomarkers to derive a composite risk score using machine-learning algorithms. The risk score is used to stratify patients and identify those who are more likely to benefit from immunotherapy.

Mezquita et al. evaluated whether pre-treatment NLR and lactate dehydrogenase (LDH) levels were associated with ICI resistance in a retrospective cohort of 466 NSCLC patients treated with PD1/PD-L1 inhibitors. Combining both parameters, the authors developed the Lung Immune Prognostic Index (LIPI). Patients with high LIPI scores (high NLR and high LDH) were correlated with the worst clinical outcomes and highest risk of disease progression [162]. The prognostic and predictive value of LIPI in ICI response was further supported by other studies [163,164,165].

Using a cohort of 99 NSCLC patients treated with ICI, Nabet et al. found that high baseline normalized bTMB, low baseline peripheral CD8 T cell level and early changes in ctDNA dynamics after a single ICI infusion were associated with durable clinical benefit. Integrating these three parameters, the authors developed a non-invasive multiparameter assay, known as DIREct-On (Durable Immunotherapy Response Estimation by immune profiling and ctDNA-On treatment) that robustly predicted patients who will achieve a durable clinical benefit (defined as PFS of at least 6 months). Patients with higher DIREct-On scores had significantly longer PFS than those with lower scores (median PFS = 8.1 months versus 2.1 months, *p* < 0.0001, HR = 7.11). DIREct-On also displayed similar classification efficacy in non-squamous or squamous NSCLC patients. Importantly, the multiparametric DIREct-On model achieved significantly better classification accuracy, outperforming each individual feature [102].

In another study of 109 NSCLC patients, Mazzaschi et al. examined pre-treatment soluble PD-L1 (sPD-L1), circulating PD1+ CD8+ and NK cells as biomarkers in predicting ICI response. Patients with high sPD-L1, low PD1+ CD8+ and low NK counts were significantly associated with poorer PFS (*p* < 0.001), OS (*p* < 0.01) and response (*p* < 0.05) to ICI. The authors further integrated these three parameters to develop an immune effector score (I_eff_S), which displayed a greater prognostic power than each individual feature [166].

These independent studies clearly demonstrated that the combined use of circulating biomarkers improved the accuracy, reliability and predictive value of biomarkers in assessing the clinical benefit of immunotherapy. In addition, circulating biomarkers may be combined with existing tumor biomarkers (e.g., TPS) to complement and further improve the prediction of immunotherapy response. The integration of these biomarkers is likely to provide more personalized care and support for individual patients.

## 5. Future Perspectives

As we move towards the era of precision medicine, liquid biopsy and circulating biomarkers are central to identifying the best possible treatment for individual patients. Although circulating biomarkers are promising tools to predict response to immunotherapy, they are not yet ready for implementation into routine clinical care. There are still challenges to clinical adoption. First, pipelines for the isolation and analysis of each circulating analyte (e.g., ctDNA, CTC, EV, immune cells) are not standardized. Various technologies exist for the extraction and enrichment of these circulating analytes and the procedures are not harmonized across laboratories worldwide. The bioinformatics tools used for analysis of specific biomarkers such as bTMB and transcriptomic signatures have not been universally applied. Second, the optimal cutoffs for patient stratification and prediction of ICI response are not universally defined. For example, some groups defined bTMB-high as ≥ 11 mutations per Mb while other groups defined bTMB-high as ≥ 16 mutations per Mb. Third, the use of NGS technology in the analysis of biomarkers (e.g., bTMB, transcriptomic signatures) would incur higher costs and longer turnaround time, which are not practical for long term clinical use. Fourth, circulating biomarkers are more applicable to patients with advanced or metastatic disease as they are more likely to shed tumor-derived cells, EVs and DNA fragments into the peripheral blood. Patients with localized tumors may not benefit from circulating biomarker analysis. Lastly, the accuracy and precision of circulating biomarkers in predicting ICI response await further validation in large prospective trials before implementation into clinical practice. In essence, the prediction of ICI response from circulating biomarker has to be sensitive, specific, reproducible and cost-effective for successful clinical adoption.

To overcome the limitations of existing circulating biomarkers, the first step is to harmonize the isolation protocols, analysis pipelines and cutoff values of each individual biomarker across laboratories worldwide. An International Consortium may have to be established to harmonize these data. The second step is to determine the predictive value of individual circulating biomarker versus the combination with other biomarkers in the context of immunotherapy response. The integrated use of biomarkers is likely to become the key strategy to improve the prediction of immunotherapy outcome. Finally, the biomarker (or combination of biomarkers) has to be validated in a multicentre large-scale prospective trial and compared with the gold standard TPS. As future research addresses these challenges, circulating biomarkers are likely to complement existing tumor biomarkers in predicting immunotherapy response.

## Figures and Tables

**Table 1:** FDA-approved immune checkpoint Inhibitors for treatment of NSCLC.

Brand Name	ICI Drug	Target	Date of FDA Approval	Stage	Line of Treatment	FDA-Approved Indications	Trials Supporting the Approval
Opdivo	Nivolumab	PD1	4 March 2015	IV	Second	Nivolumab monotherapy for metastatic squamous NSCLC patients who progressed on or after platinum-based chemotherapy or targeted therapy.	CheckMate 017 [18] and CheckMate 063 [55]
			9 October 2015	IV	Second	Nivolumab monotherapy for metastatic non-squamous NSCLC patients who progressed on or after platinum-based chemotherapy or targeted therapy.	CheckMate 057 [17]
			15 May 2020	IV	First	Nivolumab in combination with Ipilimumab for metastatic NSCLC patients with PD-L1 TPS ≥ 1% and no EGFR or ALK tumor mutations.	CheckMate 227 [56]
			26 May 2020	IV	First	Nivolumab in combination with Ipilimumab and two cycles of platinum doublet chemotherapy for metastatic or recurrent NSCLC patients with no EGFR or ALK tumor mutations.	CheckMate 9LA [57]
			4 March 2022	IB-IIIA	First	Nivolumab in combination with platinum doublet chemotherapy for early stage NSCLC patients with resectable tumors in the neoadjuvant setting.	CheckMate 816 [58]
Keytruda	Pembrolizumab	PD1	5 August 2016	IV	First	Pembrolizumab monotherapy for metastatic NSCLC patients with PD-L1 TPS ≥ 50% and no EGFR or ALK tumor mutations.	Keynote 024 [21]
			5 August 2016	IV	Second	Pembrolizumab monotherapy for metastatic NSCLC patients with PD-L1 TPS ≥ 1% at progression on or after platinum-based chemotherapy or targeted therapy.	Keynote 010 [59]
			20 August 2018	IV	First	Pembrolizumab in combination with chemotherapy (pemetrexed and platinum) for metastatic non-squamous NSCLC patients with no EGFR or ALK tumor mutations.	Keynote 189 [19]
			30 October 2018	IV	First	Pembrolizumab in combination with chemotherapy (carboplatin and either paclitaxel or nab-paclitaxel) for metastatic squamous NSCLC patients.	Keynote 407 [60]
			11 April 2019	III	First	Pembrolizumab monotherapy for stage III NSCLC patients with unresectable tumors, if PD-L1 TPS ≥ 1% and no EGFR or ALK tumor mutations.	Keynote 042 [20]
Tecentriq	Atezolizumab	PD-L1	18 October 2016	IV	Second	Atezolizumab monotherapy for metastatic NSCLC patients who progressed on or after platinum-based chemotherapy or targeted therapy.	POPLAR [61] and OAK [62]
			6 December 2018	IV	First	Atezolizumab in combination with bevacizumab and chemotherapy (paclitaxel and carboplatin) for metastatic non-squamous NSCLC patients with no EGFR or ALK tumor mutations.	IMpower150 [63]
			3 December 2019	IV	First	Atezolizumab in combination with with chemotherapy (nab-paclitaxel and carboplatin) for metastatic non-squamous NSCLC patients with no EGFR or ALK tumor mutations.	IMpower130 [64]
			18 May 2020	IV	First	Atezolizumab monotherapy for metastatic NSCLC patients with PD-L1 TPS ≥ 50% or PD-L1-positive tumor infiltrating immune cells ≥ 10% and no EGFR or ALK tumor mutations.	IMpower110 [65]
			15 October 2021	II-IIIA	Second	Atezolizumab monotherapy for stage II-IIIA NSCLC patients with PD-L1 TPS ≥ 1%, following tumor resection and platinum-based chemotherapy.	IMpower 010 [66]
Imfinzi	Durvalumab	PD-L1	16 February 2018	III	Second	Durvalumab monotherapy for unresectable stage III NSCLC patients whose disease has not progressed following concurrent platinum-based chemotherapy and radiation therapy.	PACIFIC study [67]
			10 November 2022	IV	First	Durvalumab in combination with Tremelimumab and platinum-based chemotherapy for metastatic NSCLC patients with no EGFR or ALK tumor mutations.	POSEIDON study [68]
Libtayo	Cemiplimab	PD1	22 February 2021	III	First	Cemiplimab monotherapy for advanced NSCLC patients who are not candidates for surgical resection or definitive chemoradiation therapy, if PD-L1 TPS ≥ 50% and no EGFR, ALK or ROS1 tumor mutations.	EMPOWER-Lung 1 study [69]
			8 November 2022	III–IV	First	Cemiplimab in combination with platinum-based chemotherapy for advanced NSCLC with no EGFR, ALK or ROS1 tumor mutations.	EMPOWER-Lung 3 [70]
Yervoy	Ipilimumab	CTLA4	15 May 2020	IV	First	Ipilimumab in combination with Nivolumab for metastatic NSCLC patients with PD-L1 TPS ≥ 1% and no EGFR or ALK tumor mutations.	CheckMate 227 [56]
			26 May 2020	IV	First	Ipilimumab in combination with Nivolumab and two cycles of platinum doublet chemotherapy for metastatic or recurrent NSCLC patients with no EGFR or ALK tumor mutations.	CheckMate 9LA [57]
Imjudo	Tremelimumab	CTLA4	10 November 2022	IV	First	Tremelimumab in combination with Durvalumab and platinum-based chemotherapy for metastatic NSCLC patients with no EGFR or ALK tumor mutations.	POSEIDON study [68]

**Table 2:** Advantages and limitations of circulating biomarkers.

Circulating Analyte	Biomarker	Isolation and Analytical Methods	Advantages	Limitations
ctDNA	ctDNA level Mutations DNA methylation bTMB	ctDNA isolation by magnetic beads or spin column methods. ctDNA mutation and bTMB analysis by real-time qPCR, digital droplet PCR or NGS methods. DNA methylation analysis by antibody-based methods, bisulphite sequencing or PCR-based methods.	ctDNA isolation procedures are simple, well established and do not require sophisticated device. Provides tumor-specific genetic information for molecular analysis. Allows dynamic and real-time monitoring of ctDNA levels, tumor-specific mutations and methylation status that correlate with treatment response and resistance. DNA methylation patterns are specific to cell types, which distinguish ctDNA derived from primary and metastatic sites. bTMB is an alternative for tissue-based TMB especially when tissue biopsy is too small or not available.	Short half-life (16 min–2.5 h) and low stability in circulation. Isolation and enrichment methods for ctDNA are not standardized. Low abundance of ctDNA relative to cfDNA makes detection challenging and the bioinformatics pipelines (e.g., variant calling) may produce false negatives and/or false positives. Gene panels and analysis pipelines for bTMB vary across laboratories and are not harmonized. No consensus on the optimal cutoff for ctDNA level and bTMB to predict treatment response. High cost and long turnaround time for NGS-based assays.
CTC	CTC number PD-L1 expression	CTC isolation by antibody-based (e.g., EpCAM, Cell-Search) or label-free (based on size, electric charge or deformability) microfluidics methods (e.g., ClearCell FX1, DepArray). CTC PD-L1 expression is determined by immunocytochemistry.	Provides tumor-specific information (DNA, RNA, protein) for molecular and functional analysis. Allows dynamic and real-time monitoring of CTC number and PD-L1 expression for prediction of treatment efficacy and extent of disease. CTC PD-L1 is an alternative for tumor PD-L1 especially when tissue biopsy is too small or not available.	Short half-life (1–2.4 h) and low stability in circulation. Methods for isolation and enumeration of CTC vary across laboratories and are not harmonized. Technically challenging procedures to capture intact cells for downstream analysis. Low abundance of CTC in NSCLC CTC may be derived from metastases in addition to primary tumor, thus the information derived from CTC may not be representative of primary tumor. CTC PD-L1 has no defined cutoff to predict treatment response.
Peripheral Immune Cells	PD1+ CD8+ T cell level Treg NLR TCR repertoire	Immune cells are isolated by density gradient centrifugation (e.g., ficoll, lymphoprep). Immune cell sub-populations are identified and quantified by flow cytometry. TCR repertoire is determined by NGS analysis.	Standard and reliable procedures for immune cell isolation, identification and quantification. Protocols and kits are readily available to amplify the DNA region of TCR that confers diversity and clonality. Allows dynamic and real-time monitoring of PD1+ CD8+ T cell level, Treg and NLR for prediction of treatment response and resistance.	No standardization of cutoff in immune cell level (PD1+ CD8+ T cell, Treg, NLR) for prediction of treatment response. Immune cell levels are not specific to treatment response and highly influenced by the presence of infection and inflammation. Large variations in immune cell levels among patients. No standardized procedures for TCR amplification, sequencing and bioinformatics analysis. Large differences in the accuracy and reproducibility of TCR chains, depending on the TCR profiling method used. In TCR repertoire analysis, false positive may result from clustering of functionally different clones. Lack of large-scale prospective studies.
Exosomes	Exosomal miRNA PD-L1 expression	Exosome isolation by ultra-centrifugation, sucrose gradient or microfluidics methods. Exosomal miRNA quantification by qPCR or NGS. Exosomal PD-L1 expression is determined by ELISA, immunoblot or flow cytometry.	Exosomes are abundant in the plasma. Nuclei acids are stable and protected from degradation within the exosomes. Allows dynamic and real time monitoring of exosomal RNA or protein expression for prediction of treatment response and resistance.	Isolation methods for exosomes are not standardized. Exosomes are tiny and difficult to detect (50–150 nm). Minimal overlap in the reported miRNA signatures that predict treatment response, which questions the reliability and reproducibility of these miRNA signatures. PD-L1 on exosomes may not be derived from tumor cells, could also be derived from immune or other non-tumor cells. Difficult to identify the cell type origin of exosomes (could be derived from tumor, immune or other non-tumor cells) and thus challenging to isolate pure population of tumor-derived exosomes. Quantification methods for exosomal PD-L1 expression are not standardized. Lack of large-scale prospective studies.
Plasma Proteins	Cytokines Chemokines	Protein detection and quantification by antibody-based methods (e.g., ELISA, flow cytometry, proximity extension assay).	Plasma proteins are abundant. Quantification methods are simple, sensitive and well established.	Limited stability in blood. Protein concentration is not specific to treatment response and is highly influenced by the presence of infection and inflammation. Lacks a defined cutoff to classify responders and non-responders. Lack of large-scale prospective studies.
Peripheral RNA	Plasma miRNA WBC miRNA	Total RNA isolation by spin columns or phenol-chloroform methods. miRNA quantification by qPCR or NGS.	miRNA isolation procedures are simple, well established and do not require sophisticated device. Expression of specific miRNA genes may correlate with treatment response.	No standardization for RNA isolation protocols, miRNA quantification and differential expression analysis. High cost and long turnaround time for NGS-based assays. Minimal overlap in the reported miRNA signatures that predict treatment response, which questions the reliability and reproducibility of these miRNA signatures. No defined cutoff for the expression level of miRNAs to classify responders and non-responders. Lack of large-scale prospective studies that evaluate miRNA signatures and treatment response.

Abbreviations: ctDNA, circulating tumor DNA; CTC, circulating tumor cell; bTMB, blood-based tumor mutation burden; Treg, regulatory T cell; NLR, neutrophil to lymphocyte ratio; TCR, T cell receptor; qPCR, quantitative polymerase chain reaction; NGS, next-generation sequencing; ELISA, enzyme-linked immunosorbent assay.

## Data Availability

The data presented in this study are available upon request from the corresponding author.
